# *Operando* Synchrotron X-ray Diffraction in Calcium Batteries: Insights into the Redox Activity of 1D Ca_3_CoMO_6_ (M = Co and Mn)

**DOI:** 10.1021/acs.energyfuels.1c01343

**Published:** 2021-06-16

**Authors:** A.P. Black, D. Monti, C. Frontera, D. S. Tchitchekova, R. G. Houdeville, F. Fauth, M.R. Palacin

**Affiliations:** † Institut de Ciència de Materials de Barcelona, ICMAB-CSIC, Campus UAB, 08193 Bellaterra, Catalonia, Spain; ‡ CELLS—ALBA Synchrotron, 08290 Cerdanyola del Vallès, Catalonia, Spain

## Abstract

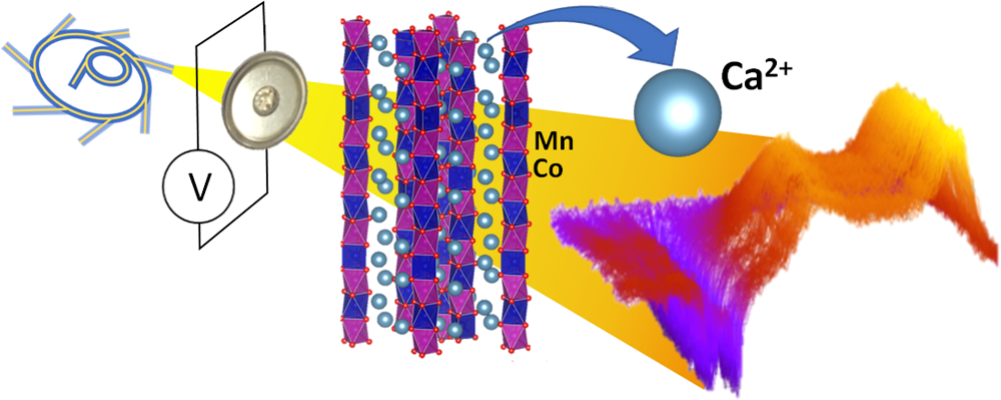

1D Ca_3_Co_2–*z*_M_*z*_O_6_ (M = Co *z* = 0, M = Mn *z* = 1, and M = Fe *z* = 0.4) were prepared and tested electrochemically. While the iron-containing phase was not found to be active, the iron- and manganese-containing phases were found to be potentially interesting as positive electrode materials for calcium metal-based high-energy battery technologies and were investigated by *operando* synchrotron X-ray diffraction. Results indicate that electrochemically driven calcium deintercalation from the crystal structure (ca. 0.7 mol per formula unit) takes place upon oxidation in both cases. The oxidized phases have incommensurate modulated crystal structures with the space group *R* 3*m*(00γ)0s and *a* = 9.127(1) Å, *c*_1_ = 2.4226(3) Å and *c*_2_ = 4.1857(3) Å, and γ = 0.579 (M = Co) and *a* = 9.217(1) Å, *c*_1_ = 4.9076(4) Å and *c*_2_ = 4.3387(4) Å, and γ = 1.139 (M = Mn), which exhibit differences due to the presence of manganese and Mn/Co ordering. The degree of calcium re-intercalation within the structure was found to be extremely limited, if any. Complementary experiments carried out in lithium cells did not show any reversibility either, thus pointing at intrinsic structural/migration constraints in the oxidized phase rather than slow kinetics of high desolvation energies associated with divalent ion charge carriers.

## Introduction

The quest for high-energy-density battery technologies has revived research on metal anode-based concepts, not only for the case of lithium, for which dendrite growth has to date been a technical showstopper, but also for other more abundant electropositive metals such as sodium, potassium, magnesium, calcium, or aluminum. Among these, the first two represent a more straightforward option, as the *know*-*how* gained in the study of lithium-based chemistries can be most valuable. In contrast, multivalent concepts come with intrinsic significant differences, which make their research more complex in nature with previous lithium-based *know-how* being less useful. Yet, theoretical energy density coupled to an expected reduced cost makes multivalent chemistries appealing.^1−3^

Despite the standard reduction potential for Ca^2+^/Ca being −2.87 versus normal hydrogen electrode, research on calcium batteries has been relatively limited until 2015.^4^ Moreover, in the absence of standards, even the development of reliable experimental protocols has required a significant effort.^5^ These are especially critical in the quest for new positive electrode materials, as the operation of calcium as the negative electrode can be plagued with a number of issues which can affect the results of positive electrode testing. For instance, electrolytes enabling calcium plating and striping are limited and not optimized.^6−9^ Hence, electrochemical response *per se* is not enough to assess the redox activity and the use of additional advanced materials characterization techniques is compulsory. In addition to that, the length scale that can be probed with such tools needs to be considered as, for instance, X-ray photoelectron spectroscopy or other surface-sensitive techniques are not trustworthy to assess bulk behavior. Finally, sample modification during handling/measurement may also lead to biased results. In this sense, *operando* diffraction is the most appealing. Besides being nondestructive, it enables capturing metastable intermediates and ascertaining the influence of operation conditions on the redox mechanism. Nonetheless, the cell geometry must be compatible with the experimental setup while at the same time being electrochemically efficient, which can be problematic in the case of new technologies due to the lack of standard cell components. The use of synchrotron radiation improves data collection statistics and angular resolution. To the best of our knowledge, synchrotron *operando* diffraction in calcium cells has only been reported for graphite electrodes at low potentials using activated carbon (AC) as the counter electrode (CE) and Ca(TFSI)_2_ in tetraglyme as the electrolyte and was helpful in assessing staging.^10^

Looking for positive electrode materials for calcium-based batteries operating at high potential, intercalation compounds appear as an appealing option. Indeed, topotactic insertion of a guest species into a host is possible for several metal ions. The host compounds should exhibit an open framework of interconnected sites through which the intercalated ion can diffuse and an electronic band structure able to reversibly accept/donate electrons. When looking for compounds able to operate as positive electrode materials, hosts containing transition metals are the most suitable, which is the reason why these are used in the ubiquitous Li-ion batteries. Within this scenario, we turned our attention toward Ca_3_Co_2_O_6_. This compound is an end member of the general A_1+*y*_(A*y*^′^B_1–*y*_)O_3_ (0 ≤ *y* ≤ 1/2) family of compounds^11^ related to the 2H (*y* = 0) hexagonal perovskite with *y* = 1/2, A = Ca, and A′ = B = Co. Its crystal structure exhibits columns of face-sharing A′O_6_ trigonal prisms and BO_6_ octahedra (in a ratio that is given by *y*) running along the *c* direction.^12−17^ This framework is similar to that of TiS_3_ where the columns consist only of trigonal prisms, and for which lithium intercalation ability has been known for long, albeit at too low potential to present a practical interest.^18^ In the case of Ca_3_Co_2_O_6_, high redox operation potential can be expected related to the oxidation of Co(III) to Co(IV) concomitant to electrochemical calcium de-insertion from the structure. Moreover, a high theoretical capacity (160 mA h/g) can be calculated considering the extraction of 1 mol of Ca^2+^ per formula unit to yield Ca_2_Co_2_O_6_. This hypothetical fully oxidized phase might correspond to CaCoO_3_, the 2H polytype of the hexagonal perovskites, which has only been reported with larger alkali earth ions (Sr, Ba) to date.^19^

In spite of such attractive performance expectations,^20^ experiments carried out under the conditions where calcium metal plating/stripping is enabled at the CE at 100 °C indicate that even if the electrochemical extraction of calcium takes place, formation of the 2H polytype is not achieved and reversibility is very limited, if any.^21^ Density functional theory (DFT) calculations for Ca_2_Co_2_O_6_ yield 0.9 eV energy barriers for the migration of calcium ions along two of the three simplest pathways considered.^22^ Upon calcium de-intercalation, trigonal prisms are expected to evolve toward distorted octahedra, which would hinder calcium re-intercalation due to a crystal field energy splitting. Moreover, calculated activation barriers higher than 2 eV are expected for a hypothetical fully oxidized 2H Ca_2_Co_2_O_6_ exhibiting the hexagonal perovskite structure.^22^ Alternative DFT studies do also point at Ca_3_Co_2_O_6_ becoming gradually less stable upon calcium extraction upon oxidation despite its chemical decomposition being likely kinetically limited.^23^ Full oxidation to Ca_2_Co_2_O_6_ has not been achieved experimentally at 100 °C, but the extraction of *ca.* 0.7 mol of calcium ions from the crystal lattice has been proved, at a potential consistent with the expected values derived from DFT calculations.^22,23^ Upon oxidation, a Ca_2.3_Co_2_O_6_ phase with an incommensurate modulated structure was formed, which does neither exhibit the composition nor the crystal structure for which migration barriers were calculated. Partial oxidation was also observed at room temperature using sodium CE and 1 M NaPF_6_ in ethylene carbonate (EC)/DMC as the electrolyte.^21^ In contrast, low capacity and no changes in the diffraction pattern of Ca_3_Co_2_O_6_ were reported for cells with calcium or tin metal CEs and Ca(TFSI)_2_-based electrolytes upon oxidation to 4 V.^23^

With the aim of fully assessing the reaction mechanism of Ca_3_Co_2_O_6_ at room temperature and getting further insights into the reversibility and/or formation of alternative phases, we performed operando synchrotron diffraction studies using a modified coin-cell setup^24^ and an alternative electrochemical configuration enabling operation at room temperature while avoiding the use of calcium metal CEs.^25^ The influence of crystal chemistry within the activity and reversibility of the system was further explored by broadening the study to other isostructural phases containing alternative transition metals which could act as redox centers. Indeed, these exhibit somewhat different cell parameters and hence different intercolumn spacings, which may have an impact on the barriers for the migration of calcium ions. The only suitable phases already reported contain iron or manganese and were previously investigated for the interest in their magnetic properties. Among these, the maximum amount of iron in the structure is achieved for Ca_3_Co_1.6_Fe_0.4_O_6_, in which Fe(III) occupies trigonal prismatic sites^26^ and has a similar unit-cell volume (741.6 Å^3^) compared to Ca_3_Co_2_O_6_ (740.9 Å^3^). For the case of manganese, Ca_3_CoMnO_6_ has been reported,^27^ in which Mn(IV) occupies the octahedral sites and exhibits a larger cell volume (764.1 Å^3^), resulting in a larger intercolumn space within the structure.

## Experimental Section

### Synthesis

Ca_3_Co_2_O_6_ was prepared by the Pechini method.^28,29^ 30 mL of a 1 M solution of Ca(NO_3_)_2_ (99% tetrahydrate, Sigma-Aldrich) and 20 mL of a 1 M solution of Co(NO_3_)_2_ (98%, hexahydrate, Sigma-Aldrich) in water were mixed with 10.5 g of citric acid (C_6_H_8_O_7_, 99%, Sigma-Aldrich) and put together in a glass vial to which 6.25 g of ethylene glycol (C_2_H_6_O_2_, 99%, Sigma-Aldrich) was added (corresponding to 3:2:5:10 molar ratios for Ca/Co/C_6_H_8_O_7_/C_2_H_6_O_2_). The solution was subsequently heated at 80 °C for 4 h and thereafter stirred overnight at room temperature to promote water evaporation, which induced enhanced viscosity of the mixture and the evolution of nitrous oxide gas. Once the gel formed dried, it was placed in an alumina crucible and heated in air first at 600 °C for 6 h and then at 950 °C for 20 h and subsequently cooled to room temperature.

The synthesis of Ca_3_Co_2-x_Fe_x_O_6_ was attempted by both the Pechini method and a solid-state reaction. In the first case, 30 mL of a 1 M solution of Ca(NO_3_)_2_ (99% tetrahydrate), 10 mL of a 1 M solution of Co(NO_3_)_2_ (98%, hexahydrate), and 10 mL of a solution of Fe(NO_3_)_3_ (98%, hexahydrate, Sigma-Aldrich) in water were mixed with 10.5 g of citric acid (C_6_H_8_O_7_, 99%, Sigma-Aldrich) and put together in a glass vial to which 6.25 g of ethylene glycol (C_2_H_6_O_2_, 99%, Sigma-Aldrich) was added (corresponding to 3:1:1:5:10 molar ratios for Ca/Co/Fe/C_6_H_8_O_7_/C_2_H_6_O_2_). Thermal treatments analogous to those enabling the synthesis of Ca_3_Co_2_O_6_ resulted in a mixture of Ca_2_Fe_2_O_5_, Ca_3_Co_2_O_6_, and/or Ca_3_Co_2–*x*_Fe_*x*_O_6_, and CaO despite a previous report suggesting the indexation of an analogous diffraction pattern with a single triclinic unit cell with no structural model provided.^30^ For the solid-state route, a stoichiometric mixture of CaCO_3_, Fe_2_O_3_, and Co_3_O_4_ was successively heated at 850, 950, and 1000 °C for 24 h in each step, with intermediate regrinding, and single-phase Ca_3_Fe_*x*_Co_2–*x*_O_6_ was achieved for *x* ≤ 0.4.

Ca_3_CoMnO_6_ was obtained both using a solid-state route^27^ (from CaCO_3_, Co_3_O_4_, and MnO_2_ after successive treatments in air at 850 °C for 20 h and 1200 °C for 24 h with intermediate regrinding) and the Pechini method^31^ (from a 3:1:1:5:10 molar ratio of Ca/Co/Mn/C_6_H_8_O_7_/C_2_H_6_O_2_ using Ca(NO_3_)_2_, Co(NO_3_)_2_, Mn(NO_3_)_2_, citric acid, and ethylene glycol with thermal treatments under synthetic air at 600 °C for 6 h and 1000 °C for 20 h).

### Characterization

Scanning electron microscopy micrographs were acquired using an FEI Quanta 200 FEG microscope under high vacuum operating at 20 kV. X-ray powder diffraction patterns were acquired in a Bruker D8 Advance A25 diffractometer in a Debye–Scherrer configuration equipped with a Mo Kα_1_ radiation source (λ = 0.7093 Å) and a Johansson monochromator. Synchrotron X-ray diffraction (SXRD) patterns were collected on MSPD beamline^32^ (ALBA synchrotron, Cerdanyola del Vallès, Spain) using the position-sensitive detector MYTHEN and λ = 0.6200 Å. In both cases, the samples, either as-prepared or recovered after running electrochemical experiments and dismantling cells inside an Ar-filled glovebox, were embedded in a 0.5 mm diameter borosilicate glass capillary and spun during data collection to ensure proper powder averaging.

### Electrochemical Experiments

Tape-casted electrodes were prepared from the mixture of as-synthesized powder samples with carbon black (Super P, Timcal, Switzerland) to enhance the electronic conductivity and polyvinylidene fluoride (Arkema) as the binder in a weight ratio of 80:10:10. The mixture was further dispersed in *N*-methyl-2-pyrrolidone (Aldrich, ≥99.9%), ground in a ball mill, casted on an 18 μm thick aluminum foil (Goodfellow, 99%) with a blade gap of 300 μm, and vacuum-dried at 80 °C for 24 h. Punched ca. 1 cm^2^ electrodes were tested in three-electrode Swagelok cells in different configurations (loading ca. 2 mg/cm^2^). Galvanostatic cycling with potential limitation (GCPL) tests were carried out at C/100 (1 mol of electrons exchanged in 100 h) at room temperature with sodium counter and reference electrodes and 1 M NaPF_6_ (Strem Chemicals, 99%) in EC/PC/DMC (0.45:0.45:0.1) as the electrolyte (all solvents, Aldrich, anhydrous, 99.0%).^33^ The interest of performing such experiments was to assess whether the electrochemical extraction of calcium was feasible using a more standard cell configuration and to provide inputs for comparison with alternative setups. Ca metal was also used as counter and pseudoreference electrodes, with 0.3 M Ca(BF_4_)_2_ dissolved in a 1:1 mixture of EC (Aldrich, anhydrous, 99.0%) and propylene carbonate (PC, Aldrich anhydrous, 99.0%) as the electrolyte, prepared from Ca(BF_4_)_2_·H_2_O salt (Alfa Aesar). Prior to electrolyte preparation, Ca(BF_4_)_2_ was dried under vacuum in a Büchi oven at 80 °C for 2 days. Once prepared, the electrolyte was further dried inside an Ar-filled glovebox with less than 1 ppm of O_2_ and H_2_O by heating at 90 °C overnight to achieve a water content lower than 80 ppm, as determined by Karl Fischer analysis. Cells were tested in the galvanostatic mode at C/200 (defined here as the reaction of 1 mol of Ca^2+^ in 200 h and equivalent to 1 mol of electrons exchanged in 100 h) on a Bio-Logic VMP3 potentiostat either at room temperature, 100, or 115 °C. The stability of the active materials under the experimental conditions was ascertained by ensuring that their XRD patterns were unmodified after keeping cells at open-circuit potential for 3 h at the temperature of testing.

Powder electrodes were prepared by simply mixing active materials with carbon black (Super P, Timcal, Switzerland) in a weight ratio of 75:25, respectively. *Operando* electrochemical tests were performed in two types of *in situ* cells: drilled 2032 coin cells with a 4–5 mm glass window^24^ and Leriche-type cells.^34^ An aluminum foil (3 μm, Goodfellow) was placed on the positive electrode side to protect the window and ensure good electric contact. One glass fiber filter disk (Whatman, GE Healthcare, 420 μm thick) was used as a separator. Electrochemical experiments were carried out in the galvanostatic mode at room temperature and C/50 (defined as the reaction of 1 mol of Ca^2+^ in 50 h) using the same electrolyte as described above. AC cloth (Kynol, ACC-509220) was used as the CE. The advantages of using AC include high reversibility, operation window matching the stability of most electrolytes, versatility in terms of ions that can be adsorbed, and operation temperature. Yet, its relatively low specific capacity (35 mA h/g at 25 mA/g, between 0 and −1.4 V vs Ag pseudoreference electrode)^25^ requires careful cell balancing to ensure largely oversizing the capacity of the active material tested. Therefore, coin cells were loaded with 2 mg of active material, 0.3 mL of electrolyte, and 3 AC fabric discs of 10 mm in diameter, reaching a total mass of 37 mg at the CE side. Leriche-type cells were loaded with 6 mg of active material and 3 AC fabric discs of 14 mm diameter and a total mass of 66 mg. In the case of the drilled coin cells, *operando* measurements were conducted at ALBA synchrotron on the powder diffraction station of the MSPD beamline with patterns being collected in the ≈2.3 ≤ 2θ ≤ 48° range in 0.006° steps and an integration time of 172 s. The Leriche-type cell was used to conduct *operando* experiments on a Bruker D8 Advance A25 diffractometer in a Bragg–Brentano configuration equipped with a Cu Kα_1,2_ radiation source (λ = 1.5406 and 1.54443 Å). The XRD patterns were collected in the 10 ≤ 2θ ≤ 50° range in 0.02° steps and an integration time of 2 h.

## Results and Discussion

### Synthesis and Electrochemical Characterization

**Figure 1:**
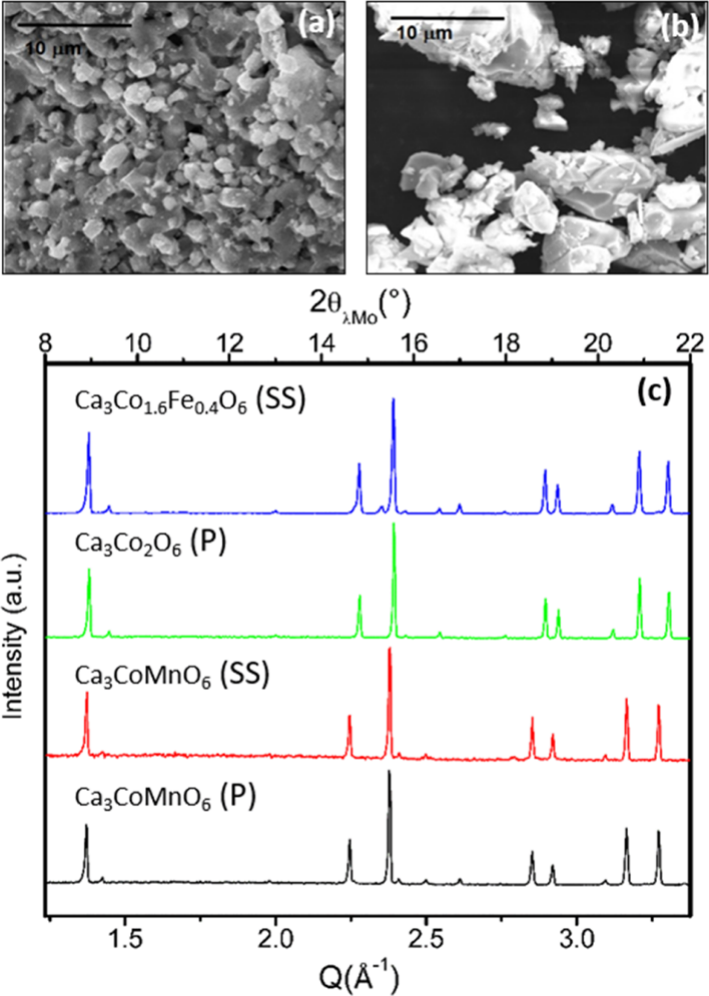
Representative scanning electron micrographs for samples resulting from Pechini synthesis ((a) corresponding to Ca_3_Co_1.6_Fe_0.4_O_6_) and the solid-state reaction ((b), Ca_2_CoMnO_6_) and (c) XRD patterns (λ_Mo_) of as-prepared compounds Ca_3_Co_1.6_Fe_0.4_O_6_ SS, Ca_3_Co_2_O_6_ P, Ca_3_CoMnO_6_ SS, and Ca_3_CoMnO_6_ P (SS denotes solid-state synthesis and P stands for the Pechini method).

Pure Ca_3_Co_2_O_6_, Ca_2_CoMnO_6_, and Ca_3_Co_2.6_Fe_0.4_O_6_ were obtained using the conditions reported in the Experimental Section. Figure 1 depicts the corresponding XRD patterns and representative scanning electron micrographs. As expected, the Pechini method delivered more homogeneous and smaller (<5 μm) particles than solid-state reactions.

Rietveld refinements indicate that the prepared compounds are free from impurities (except for Ca_3_CoMnO_6_ prepared by the solid-state reaction, in which some low-intensity impurity peaks are clearly visible in the pattern corresponding to 3% CaO as deduced from the refinement). Since previous neutron powder diffraction studies have shown that both Mn and Fe replace Co in the octahedral position,^27,28^ we have presumed this ordering of transition metals to take place. All patterns have been successfully refined using the *R*3̅*c* space group and the structure involving the alternation of face-sharing MO_6_ octahedra CoO_6_ trigonal prisms (M = Fe and Mn). Occupancies at octahedral and prismatic sites, constrained by the overall stoichiometry and the full occupancy of both sites, are consistent with the model used. Refined cell parameters also agree with those reported in the literature: *a* = 9.0771(2) Å and *c* = 10.3804(2) Å (Ca_3_Co_2_O_6_); *a* = 9.1296(2) Å and *c* = 10.5810(2) Å (Ca_3_CoMnO_6_, solid-state reaction); *a* = 9.1295(2) Å and *c* = 10.5822(2) Å (Ca_3_CoMnO_6_, Pechini method); and *a* = 9.0770(2) Å and *c* = 10.3803(2) Å (Ca_3_Co_1.6_Fe_0.4_O_6_).

**Figure 2:**
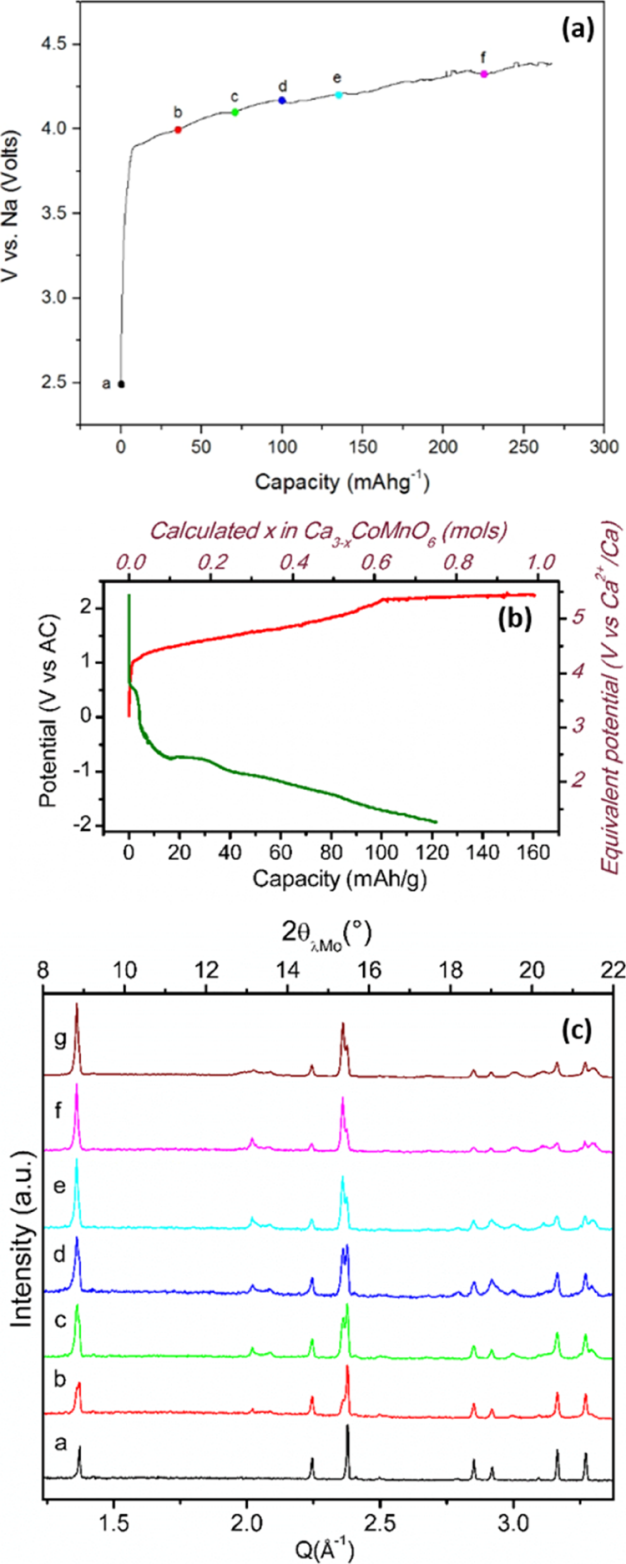
Potential vs capacity profiles for Ca_2_CoMnO_6_ composite electrodes oxidized in cells at room temperature (a) using sodium reference and counter electrodes and (b) Ca(BF_4_)_2_ in the EC/PC electrolyte at RT at C/50. (c) Evolution of the *ex situ* XRD patterns (λ_Mo_) of Ca_2_CoMnO_6_ when oxidized in electrochemical cells at room temperature vs sodium CEs (1 M NaPF_6_ in EC/PC/DMC as the electrolyte), color codes are related to the top figure for patterns (a–f). Pattern g corresponds to an electrode fully oxidized in a calcium cell.

The feasibility of electrochemical oxidation on iron- and manganese-containing compounds was tested at room temperature in cells using sodium counter and reference electrodes and sodium-based electrolytes, in conditions which had previously enabled the oxidation of Ca_3_Co_2_O_6_ concomitant to Ca^2+^ extraction.^21^Figure 2A depicts the potential versus capacity profile of Ca_3_CoMnO_6_ composite electrodes upon oxidation in sodium cells. The potential increases rapidly to 3.9 V versus Na^+^/Na and then a sloping region is observed, with a capacity of 267 mA h/g being achieved at 4.4 V. This behavior is similar to the one of Ca_3_Co_2_O_6_, with most likely some electrochemical capacity being related to electrolyte decomposition at high potential. Analogous experiments attempting to oxidize Ca_3_CoMnO_6_ were carried out in calcium cells using AC as the CE and a silver wire as the pseudoreference electrode. Figure 2B depicts the characteristic potential versus capacity profile during a single oxidation/reduction galvanostatic cycle using 0.3 M Ca(BF_4_)_2_ in EC/PC as the electrolyte (54 ppm H_2_O) at the C/50 rate. The oxidation evolves through a pseudoplateau centered around 1.5 V versus AC with an associated capacity of 100 mA h/g. Further oxidation involves a linear increase in potential up to 2.1 V (160 mA h/g). Upon reduction, the potential decreases rapidly down to −1.2 V versus AC and a sloping region is further observed down to −2 V versus AC (corresponding to a capacity of 120 mA h/g). Figure 2C depicts the XRD patterns of the (a–f) samples shown in Figure 2A oxidized to different extents in sodium cells and an additional pattern (g) oxidized in a calcium cell (200 mA h/g) at room temperature in 0.3 M Ca(BF_4_)_2_ in EC/PC using Ca metal as counter and reference electrodes. Upon oxidation, patterns gradually evolve with peaks at *Q* = 1.37, 2.23, and 2.37 Å^–1^ of the pristine phase decreasing in intensity with their position remaining unchanged and new (strong) peaks appearing at 2.95 and 1.36 Å^–1^, together with some tiny peaks in the region of *Q* ∼ 2 Å^–1^. Thus, regardless of the CE and electrolyte used, the changes in the diffraction pattern observed upon oxidation are the same and hence attributed to electrochemical calcium extraction from the crystal structure to form Ca_3–*x*_MnCoO_6_, similar to what had been observed for Ca_3_Co_2_O_6_.^21^

Attempts to oxidize Ca_3_Co_1.6_Fe_0.4_O_6_ were unsuccessful, as no changes were observed in the XRD patterns despite a plateau being observed at a high potential (3.9 V vs Ca^2+^/Ca) with a capacity close to 200 mA h/g, which was thus attributed to electrolyte decomposition. In this case, one must conclude that any electrochemical activity for this compound, if existing, takes place beyond the electrochemical stability window of the electrolyte used.

### *Operando* Diffraction Studies

*Operando* synchrotron diffraction experiments involving materials for calcium batteries are challenging. Beyond the general requirement of compatibility of all the components that are integrated in the *in situ* cells in terms of corrosion stability, the sluggish diffusion rates of the calcium ion into the crystal host material impose a limitation in terms of C-rate and hence unavoidably long experiments. Finally, the lack of electrolytes enabling calcium plating/stripping at room temperature and a fast C-rate which are also stable at high potentials (>4 V *vs* Ca^2+^/Ca) constitutes a showstopper for these materials.

**Figure 3:**
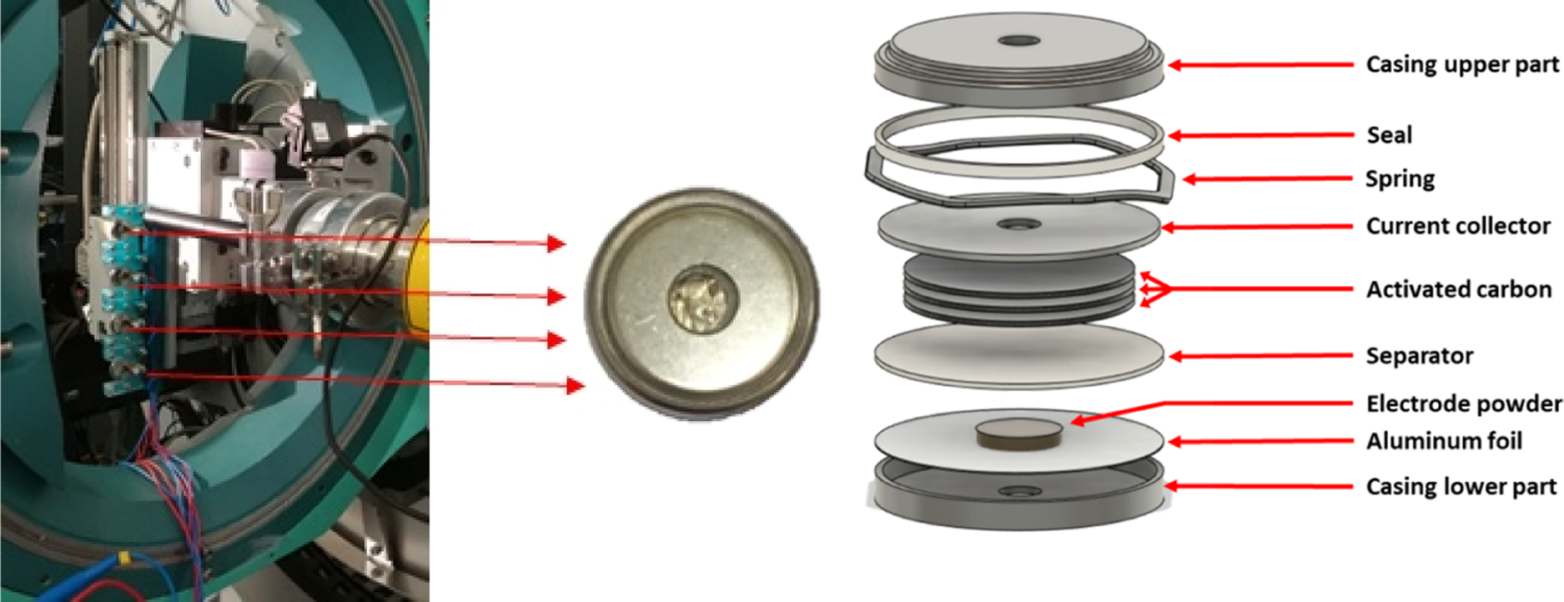
Sample holder with four coin cells to be simultaneously monitored *operando*, single coin-cell image, and an exploded view of a coin cell.

In the present study, and in order to enhance the efficiency of the full experiment, a setup enabling the alternative sequential measurement of four cells was used (see Figure 3) inspired in ref (24).

*Operando* experiments aiming at getting further insights into the redox activity of 1D Ca_3_CoMO_6_ (M = Co and Mn) were carried out using 0.3 M Ca(BF_4_)_2_ in EC/PC as the electrolyte (54 ppm H_2_O) and consisted of a single oxidation/reduction cycle at C/50 within an electrochemical potential window between 2.5 and −2.5 V versus AC. Since the main objective was to assess the reversibility of the process, if any, the switch between oxidation and reduction steps was made manually once there was clear evidence of significant calcium extraction. Since C/50 is a rather slow rate, in order to optimize the beamtime, some cells were cycled *ex situ* and placed under the beam to be monitored only when they reached a certain state of charge. This also enabled us to assess the reproducibility of the redox behavior both under and outside the beam.

**Figure 4:**
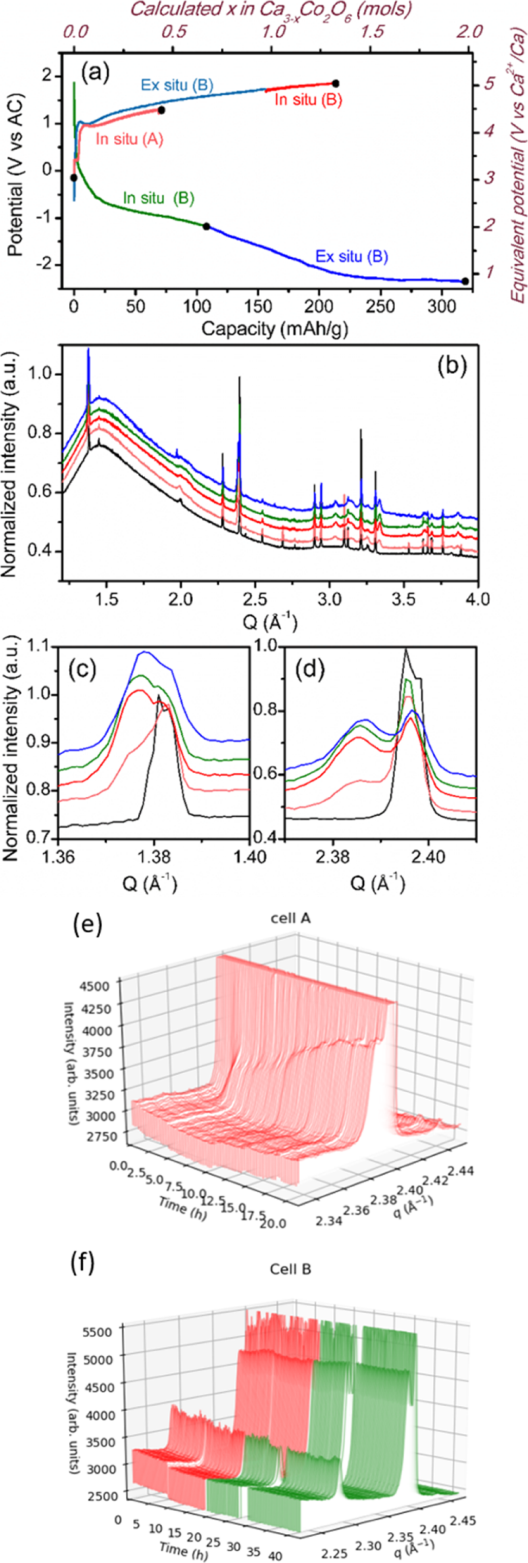
Potential vs capacity profiles from GCPL experiments on Ca_3_Co_2_O_6_//AC cells using Ca(BF_4_)_2_ in EC/PC as the electrolyte at RT and C/50 rate (a), corresponding SXRD patterns (b) and zoom-in images (c,d), selected patterns correspond to the first and last pattern of cell (A) in black and light red, last oxidation and last reduction of cell (B) in red and dark green, and last ex situ pattern cell (B) in blue. (e,f) Zoom-in images of reflections 100 and 300 of in situ SXRD patterns of cells (A,B), respectively.

In the case of Ca_3_Co_2_O_6_, different cells were monitored *operando* starting at different states of charge. Cell A was launched to cover the initial oxidation up to a specific capacity of 52 mA h/g. Cell B was initially oxidized out of the beam (160 mA h/g, equivalent to 1 mol of Ca^2+^ extracted if this was the only redox process taking place, see the upper abscissa axis in Figure 4a) and placed under the beam for further oxidation up to 213 mA h/g, followed by a reduction step. The electrochemical curve evolves through a pseudoplateau centered at about 1.5 V versus AC upon oxidation, and a more sloping trend upon reduction, centered at ca. −1.2 V versus AC. Selected *in situ* SXRD patterns for those cells displayed in Figure 4b–f are in full agreement with the results from previous experiments using the same electrolyte but carried out at 100 °C and using calcium metal CEs.^21^ The (300) and (100) Bragg reflections, at *Q* = 2.396 and 1.383 Å^–1^, respectively, progressively decrease in intensity, whereas new peaks grow at *Q* = 2.386 and 1.377 Å^–1^. The latter are ascribed to Ca_3–*x*_Co_2_O_6_ with *x* ∼ 0.7, which exhibits an incommensurate modulated crystal structure [space group *R* 3*m*(00γ)0s, *a* = 9.127(1) Å, *c*_1_ = 2.4226(3) Å and *c*_2_ = 4.1857(3) Å, and γ = 0.579(2)]. The Ca content in this phase is straightforwardly related to the propagation vector, determined by the position of the tiny peak appearing at *Q* ∼ 2 Å^–1^ among others.^12,21^ The patterns depicted in Figure 4b–f correspond to pristine Ca_3_Co_2_O_6_, intermediate, and final stage of oxidation as well as the intermediate and final stage of reduction. From the evolution of patterns represented in Figure 4e, a progressive intensity increase for the (100)′ reflection at *Q* = 1.377 Å^–1^ as a function of oxidation time can be inferred. The patterns observed at the end of oxidation are consistent with the electrodes containing a mixture of both oxidized Ca_3–*x*_Co_2_O_6_ and pristine Ca_3_Co_2_O_6_ phases. No significant evolution of the peaks, neither in position nor in intensity, is seen when the polarity of the cell is reversed. Indeed, Figure 4f depicts the patterns taken upon reduction and no changes are evidenced in the region of (300)′ (*Q* = 2.386 Å^–1^) throughout the process (the last pattern corresponds to a capacity of 325 mA h/g). Such results are fully consistent with those derived from diffraction patterns taken *ex situ* after electrochemical experiments were carried out at 100 °C and using Ca metal as the CE and indicate that the electrochemical extraction of calcium from the crystal structure is irreversible in the experimental conditions used and the capacity recorded is only related to side reactions involving the electrolyte.

**Figure 5:**
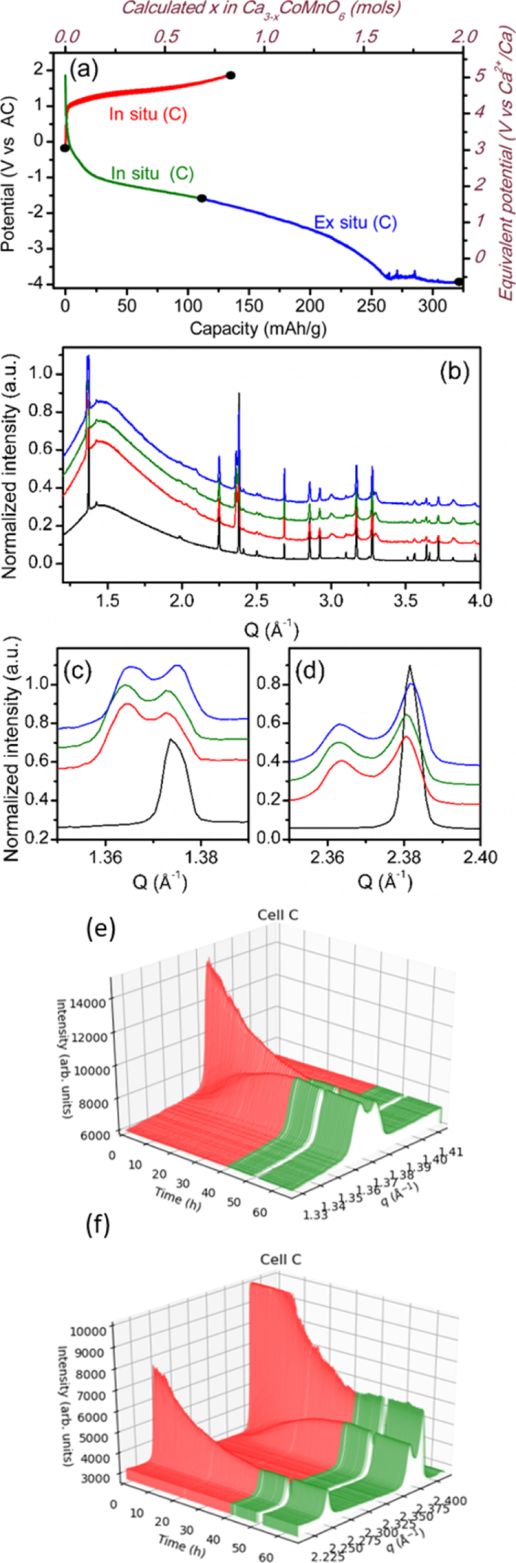
Potential vs capacity profiles from GCPL experiments on Ca_3_CoMnO_6_//AC cells using Ca(BF_4_)_2_ in EC/PC as the electrolyte at RT and C/50 (a), corresponding SXRD patterns (b) and zoom-in images (c,d), selected patterns correspond to pristine and last pattern in oxidation in black and red, last reduction in dark green, and last ex situ pattern of cell (C) in blue. (e,f) Zoom-in images of reflections 100 and 300 of in situ SXRD patterns, respectively.

Figure 5a displays the characteristic potential versus capacity profiles for Ca_3_CoMnO_6_ electrodes during galvanostatic testing at C/50 in the course of the *operando* experiment. The red curve corresponds to the oxidation step, which evolves through a pseudoplateau around 1.3 V versus AC [equivalent to 4.5 V vs Ca^2+^/Ca (see the right axis in Figure 5a)], up to a capacity of about 133 mA h/g that would correspond to a nominal extraction of 0.82 mol of Ca^2+^ from the crystal structure (top abscissa axis in Figure 5a). The reduction step is denoted in green for the section monitored under the beam (*operando*) to a capacity of 112 mA h/g and in blue beyond that value, with a final diffraction pattern taken after extended reduction to a capacity of 325 mA h/g (black dot). Oxidation evolves through a pseudoplateau centered at about 1.3 V versus AC. The reduction process proceeds through a sloping region down to −3.8 V versus AC and ca. 260 mA h/g, after which noisy behavior is observed, probably due to bubbling related to electrolyte decomposition.^35^

The SXRD patterns collected throughout the experiment are shown in Figure 5b. Zoom-in images (c–f) highlight the regions where the evolution of the phase composition is clearly observed, with red patterns corresponding to oxidation and green patterns to reduction. Upon oxidation of Ca_3_CoMnO_6_, a progressive decrease in the intensity of the peaks at *Q* = 1.365 and 2.363 Å^–1^ occurs at the expense of new peaks appearing at *Q* = 1.375 and 2.382 Å^–1^ [zoom-in images (d) and (e)] and also at *Q* = 2.03, 2.09, 3.00, and 3.30 Å^–1^, which gradually grow upon oxidation. These are consistent with the formation of a new Ca_3–*x*_CoMnO_6_ phase upon oxidation, in agreement with the results of *ex situ* experiments (Figure 2c).

**Figure 6:**
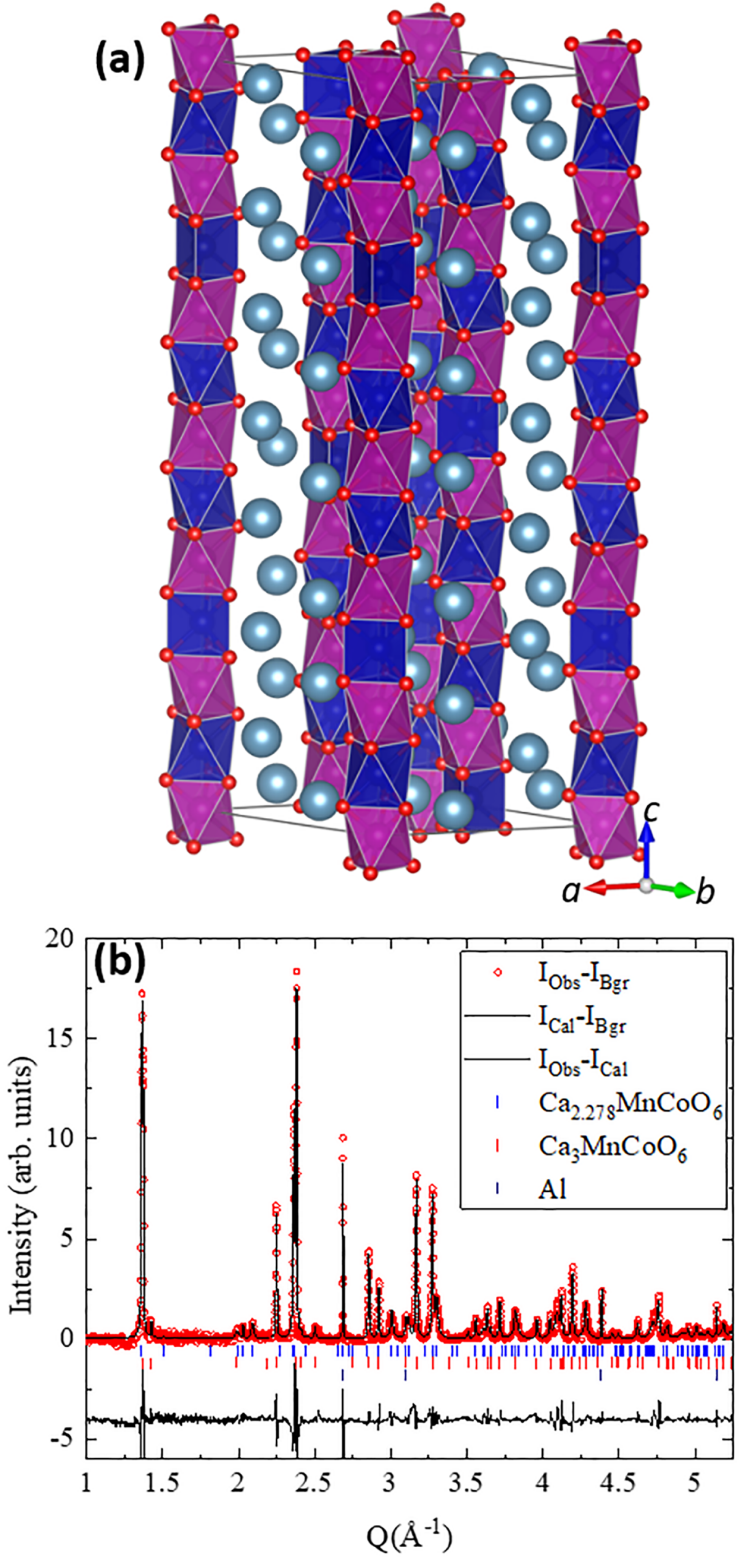
(a) Crystal structure model for Ca_2.28_MnCoO_6_ (Co is placed at the center of the dark blue polyhedra and Mn at the center of magenta octahedra, and oxygen atoms are represented by small red spheres and Ca by light blue, large spheres) showing the proposed incommensurate modulation. (b) Rietveld refinement of the crystal structure based on the diffraction pattern using the *R*3*m*(00γ)0s structure plus a remaining fraction of the pristine Ca_3_CoMnO_6_ phase and a small signal coming from the Al foil.

The patterns corresponding to Ca_3–*x*_CoMnO_6_ bear great similarity with those corresponding to Ca_3–*x*_Co_2_O_6_ (*x* ∼ 0.7). Peaks (100) and (300) progressively decrease, while reflections attributed to Ca_3–*x*_CoMnO_6_ appear at slightly lower *Q*-values (Figure 5C,D) than for Ca_3–*x*_Co_2_O_6_. In addition to those, other low-intensity peaks appear, which are not consistent with the incommensurate structure of Ca_3–*x*_Co_2_O_6_. This does not come as a surprise because despite pristine compounds being isostructural, the incommensurate structure of Ca_3–*x*_Co_2_O_6_, with only one Co crystallographic position,^12,13^ is not compatible with the Mn/Co ordering present in Ca_3–*x*_CoMnO_6_. Indeed, in the incommensurate structure of Ca_3–*x*_Co_2_O_6_, any transition metal position is suitable to switch (when varying the Ca content) from octahedral to prismatic, but it is unlikely that Mn would occupy a prismatic position. According to neutron powder diffraction (and our own refinements), Mn–O bond distances are close to 1.905 Å^28^ and consistent with Mn^4+^, far from the bond distances observed in prisms (>2.1 Å). Thus, an alternative incommensurate crystal structure model (Figure 6a) has been built, which is compatible with the Co/Mn order while keeping Mn in the octahedral environment and is detailed in the Supporting Information.

The Rietveld refinement of the last pattern collected upon oxidation using this model is plotted in Figure 6b (background has been subtracted for clarity). The refined values of the cell parameters are *a* = 9.217(1) Å, *c*_1_ = 4.9076(4) Å, and *c*_2_ = 4.339(3) Å (γ = 1.139) for the incommensurate phase [48(2) wt %] and *a* = 9.150(1) Å and *c* = 10.596(1) Å for the pristine phase [52(2) wt %]. The calcium content can be deduced from the value of the propagation vector and is consistent with Ca_2.28_CoMnO_6_. At the end of oxidation, Ca_2.28_CoMnO_6_ represents about 48(2) wt % and is hence equivalent to 0.35 mol of Ca^2+^ extracted per formula unit. This value is significantly smaller than the one estimated from the electrochemical capacity (0.82), which indicates that partial contribution to the current comes from a parasitic redox process, most likely related to electrolyte decomposition. The reasons for the extraction of a lower amount of calcium for this compound are not clear at this stage. It could be speculated that the ordering of Co and Mn in Ca_3_CoMnO_6_ makes calcium deinsertion entropically less favorable with respect to that of Ca_3_Co_2_O_6_, as the position of pyramidal environments is restrained to Co sites. Moreover, the migration mechanism for calcium ions (see the Supporting Information) implies the simultaneous rotation of two oxygen triads versus one single rotation in the case of Ca_3_Co_2_O_6_.

Rietveld refinements of the pattern taken at the end of reduction indicate that despite a slight growth of (100) and (300) peaks at *Q* = 1.374 and 2.381 Å^–1^, the amount of Ca_2.28_CoMnO_6_ is still 42(3)wt %, which would point at only 0.04 mol of Ca^2+^ ions being reinserted in the crystal structure upon reduction. Moreover, this result must be taken with caution as the precise region of the sample where the measurement is done could not be exactly the same after relocating the cell under the beam.

To further assess whether the limited reversibility observed, if any, is related to the sluggish difusion of Ca^2+^ ions within the oxidized phase or the strong Ca^2+^–solvent interactions, causing high desolvation energies which may hinder calcium reintercalation, or related to electrolyte decomposition, resulting in the formation of some blocking films on the surface of the particles, an additional operando experiment was conducted in lithium cells. In this case, standard electrolytes known to be stable at high potentials are used. The charge compensation mechanism during the oxidation of Ca_3_CoMnO_6_ should also be related to the extraction of calcium ions from the crystal structure, while upon reduction, the lithium ions present in the electrolyte (much more mobile than calcium ions) might be inserted in the crystal structure.

**Figure 7:**
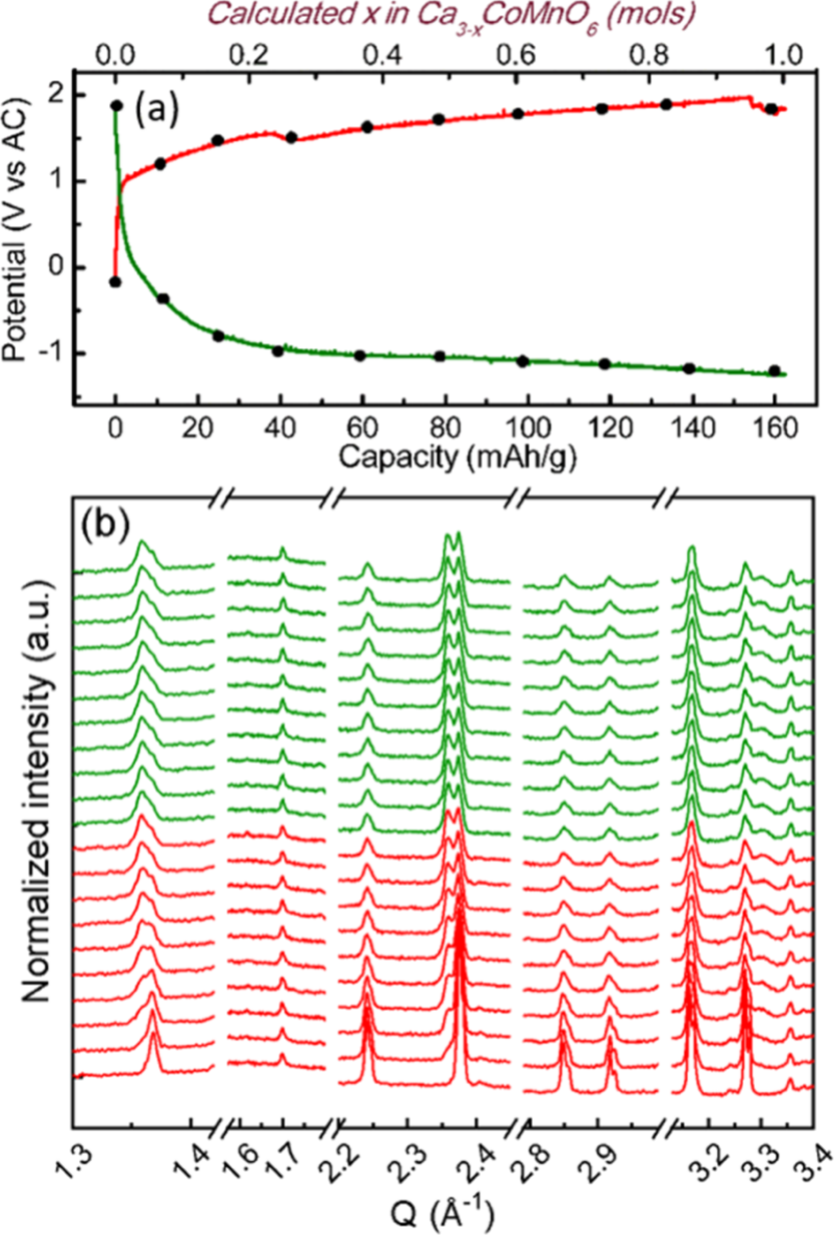
Potential vs capacity profiles from the GCPL experiment on the Ca_3_CoMnO_6_//AC cell using 1 M LiPF_6_ in EC/DMC as the electrolyte at RT and C/50 (a) and corresponding selected XRD patterns (b).

Figure 7a displays the characteristic potential versus capacity profiles of Ca_3_CoMnO_6_ electrodes during operando galvanostatic oxidation and reduction at C/50 with 1 M LiPF_6_ in EC/DMC as the electrolyte and using AC as the CE. The potential versus capacity profiles upon oxidation in lithium and calcium cells are similar, while the reduction takes place at a slightly higher potential for the case of lithium, which may be related to the different ionic conductivities of the electrolytes inducing different cell polarizations. Representative operando diffraction patterns taken in lithium cells are depicted in Figure 7b. Upon the oxidation of Ca_3_CoMnO_6_, the evolution observed is fully consistent with that observed in calcium cells, reaching a stage in which both pristine and oxidized phases still coexist for a capacity of 160 mA h/g. Upon reduction, no changes in the patterns are evidenced, which forces us to conclude that the capacity observed is related to side reactions involving the electrolyte. These results point at the irreversibility of the redox process being related to intrinsic structural/migration constraints rather than to slow kinetics or high desolvation energies. Calcium ion rearrangement in the oxidized phase sublattice may hinder mobility in Ca_3–*x*_CoMnO_6_, as suggested for Ca_3–*x*_Co_2_O_6_.^22^ Indeed, oxidation of Ca_3_CoO_6_ and Ca_3_CoMnO_6_ does not follow a topotactic mechanism and it is reasonable to assume that the phase transformation that gives rise to the formation of the incommensurate modulated structure induces changes in the Ca migration pathways, with the intercolumn space not making any significant difference.

## Conclusions

The *operando* synchrotron diffraction experiments indicate that the extraction of Ca^2+^ from K_4_CdCl_6_-type Ca_3_CoMO_6_ (M = Co, Fe, or Mn) structures is achieved at room temperature for M = Co and Mn only. Different incommensurate modulated structures are formed upon oxidation for M = Co and M = Mn, as a result of Mn/Co ordering, with the propagation vector enabling the direct estimation of the calcium content, which is similar for both oxidized phases (ca. 2.3 mol per formula unit). The reversibility of the redox process was found to be extremely limited, if any. Further *operando* experiments performed in lithium cells also resulted in the electrochemical extraction of calcium from the crystal structure upon oxidation, but intercalation of Li^+^ in Ca_3–*x*_CoMnO_6_ upon reduction was not found to be feasible either. Thus, the main factor behind the absence of redox reversibility seems to be related to intrinsic structural/migration constraints rather than to slow kinetics or high desolvation energies commonly associated with divalent ion charge carriers.

Finally, although still not conventionally used in the emerging field of calcium batteries, we believe that the experimental protocols reported herein can be generally extended to the screening of new multivalent ion positive electrode materials and be a valuable tool to unambiguously assess reactivity and reversibility.

## Supporting Information Available

Proposed description of the incommensurate structure of Ca_3–*x*_CoMnO_6_ (PDF)
