# Supplementary material for: Operando Synchrotron X-ray Diffraction in Calcium Batteries: Insights into the Redox Activity of 1D Ca3CoMO6 (M = Co and Mn)
Source: Energy Fuels. 2021 Jun 16;35(13):10898–907. doi: 10.1021/acs.energyfuels.1c01343 (PMC8279016; doi:10.1021/acs.energyfuels.1c01343)
Supplement: Supplementary file 1 — ef1c01343_si_001.pdf [file ef1c01343_si_001.pdf]

Operando synchrotron X-ray diffraction in calcium batteries: insights into redox activity of 1D Ca_3CoMO_6 (M=Co, Mn)

A.P. Black,[1] D. Monti,[1] C. Frontera,[1] D. S. Tchitchekova,[1] R. G. Houdeville,[2] F. Fauth,[2] M.R. Palacin[1,*]

[1] Institut de Ciència de Materials de Barcelona, ICMAB-CSIC, Campus UAB, 08193 Bellaterra, Catalonia, Spain.

[2] CELLS - ALBA synchrotron, 08290 Cerdanyola del Vallès, Catalonia, Spain

Proposed description of the incommensurate structure of $Ca_{3-x}CoMnO_6$

To the best of our knowledge, there is no previous work describing the incommensurate structure of $Ca_{3-x}CoMnO_6$ for an arbitrary x value. By the Ca extraction from $Ca_3Co_2O_6$, the resulting compound ($Ca_{3-x}Co_2O_6$) can be described in general as belonging to the family $A_{1+y}(A'_yB_{1-y})O_3$ ($y \leq 0.5$). [1,2]
Ca extraction from $Ca_3Co_2O_6$ is accompanied by the rotation (by about 60º around 001 direction) of trios of oxygen atoms forming a prism-octahedra shared face that "dynamically" convert prisms into octahedra and, in that way "displace" the prisms. This is illustrated in figure S1a. In terms of description, incommensurate composite structure is formed by two subsystems (see e.g. [1,3]). First one is CoO_3, and the second one is Ca_{1+y}. They are described by two different *c*-cell parameters (c_1 and c_2), and the same *a* parameter (crystal system is hexagonal). c_1 corresponds to the average Co-Co distance and c_2 to twice the average Ca-Ca distance. Thus, Ca/Co ratio is determined by $\gamma=c_1/c_2$ ($1+y=2\gamma$). Each Co-polyhedron shared-face is described by means of six oxygen atoms (two sets of three oxygen atoms related between them by a rotation of about 60º around 001 direction), with different x_4 coordinate. By using Crenel functions for their occupancies, the difference in the fourth coordinate makes that only three of the six are present (their occupancies are 1, while for the other three they are 0) and allows the model to alternate between octahedra and prisms according to the incommensurability (x_4 follows the periodicity imposed by c_2 that is incommensurate with respect to c_1). During the oxidation of $Ca_3Co_2O_6$ the amount of Ca varies "continuously" (see for instance figure 2d in [2]), so it does c_2 and the position of CoO_6 octahedra and prisms. As it is c_2 what determines the distribution of the two types of polyhedral, their fractions are directly governed by the amount of Ca rendering the initial formula $A_{1+y}(A'_yB_{1-y})O_3$ with A= Ca, A'=Co (in prisms), and B=Co (in octahedra).
Many works have studied the structure of compounds having different species for A' and B sites, but all of them belong to these series and thus the A'-B stoichiometry is directly related to A-stoichiometry and, in all these studies, this is a fixed parameter. In these cases then, A' are comfortably placed in prismatic coordination while B in octahedral ones with no interchange between them. This is described, in terms of the incommensurated structure, by using Crenel functions for the occupancies of A' and B that turn them from 1 to 0 and viceversa according to the value of x_4 and following the periodicity imposed by c_2 [4]. Thus, somehow we can imagine that the case of the structures of the type $A_{1+y}(A'_yB_{1-y})O_3$ with two different chemical species for A' and B

is a “snapshot” of the “dynamical” Ca extraction, in which Co in prisms corresponds to A’ and Co in octahedra to B (in this notation y ranges between 0 and 0.5).
The situation that is induced by the Ca extraction in $Ca_{3-x}CoMnO_6$ is then considerably different as in the Co only case or in the general $A_{1+y}(A'_yB_{1-y})O_3$ case as the Co-Mn stoichiometry is no longer related to the Ca content and the analogous arbitrary rotation of the prism-octahedron shared faces would induce Mn in prismatic environment that is very unlike as explained in the main text. Moreover, as the pristine compound presents Mn/Co cationic order, the extracted structure must present this order.

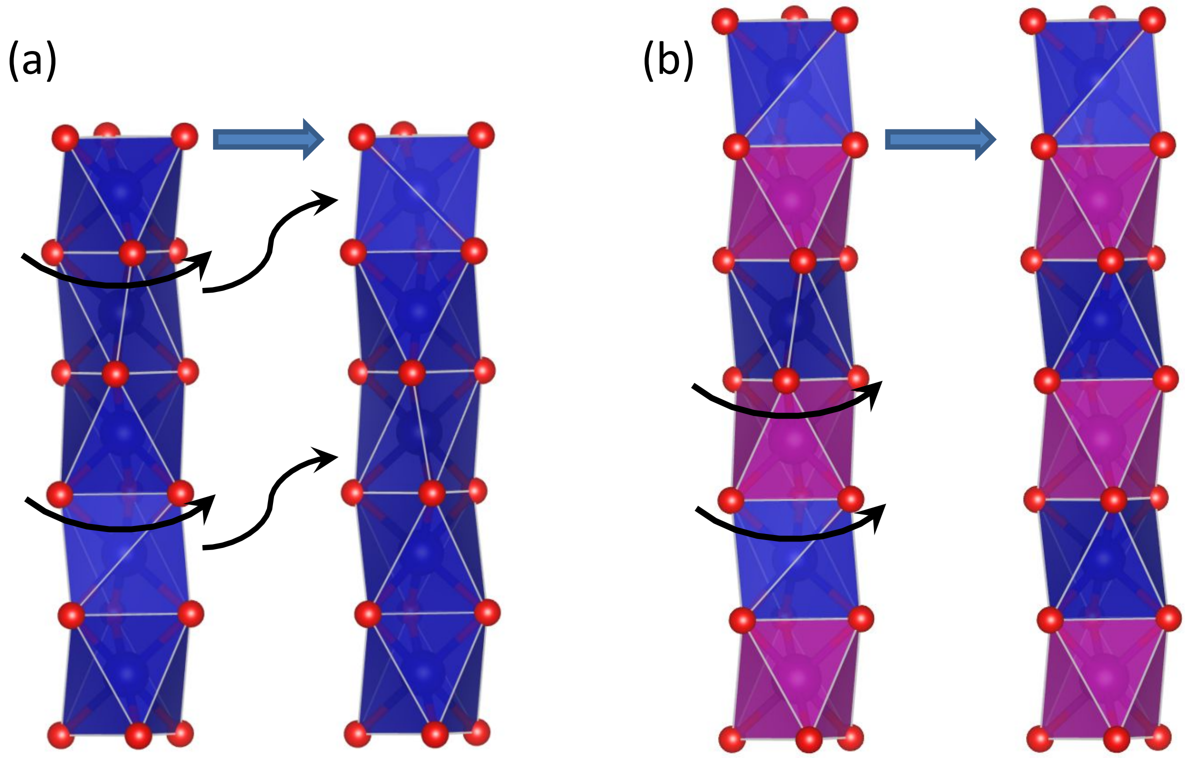

Figure S1. Simplified picture of the mechanism for (a) displacing CoO_6 prisms in $Ca_{3-x}Co_2O_6$ (b) or turning them into octahedra in $Ca_{3-x}MnCoO_6$, induced by the extraction of Ca in $Ca_{3-x}CoMO_6$ (M=Co, Mn).

It is out of our scope to try to solve and refine the unknown (up to the best of our knowledge) incommensurate phase formed by this mechanism because single crystal data of good quality would be needed for this purpose. Our data, instead, have all the inconveniences inherent to an in-situ experiment (large background, limited signal to noise ratio, the possibility of insufficient grain averaging, etc.), consequently such a task is unreliable. Nonetheless, in order to be able to estimate the amount of Ca extracted from the system, we have constructed a model that fulfils the requirements mentioned and that is able to index the tiny incommensurate diffraction peaks appearing during oxidation. The model is compatible with the Mn/Co ordering and able to accommodate the continuous transformation of CoO_6 prisms into CoO_6 octahedra without altering MnO_6 octahedra [beyond rotating them as a whole, as illustrated in figure S1(b)]. The model is intended to be a minimal modification of that describing $Ca_{1+y}CoO_3$. In analogy, it consists of two subsystems, one for Mn-Co-O columns and the other one for Ca. Both parts have the same *a* lattice parameter and different *c* parameter (c_1 for

$MnCoO_6$ subset and c_2 for Ca one). Thus, the propagation vector, as in $Ca_{3-x}Co_2O_6$ case, is of the type (00γ). Now c_1 is about twice that of $Ca_{3-x}Co_2O_6$ (it also corresponds to Co-Co distance, but this distance is doubled because of the Mn in between). Doubling this c_1 parameter allows the introduction of two different crystallographic sites in block 1 (Co and Mn) keeping the Co-Mn ordering. In this model, the transformation between prisms and octahedra takes place by rotating a whole MnO_6. This transforms the surrounding CoO_6 prisms into octahedra by keeping the Mn in octahedral environment.

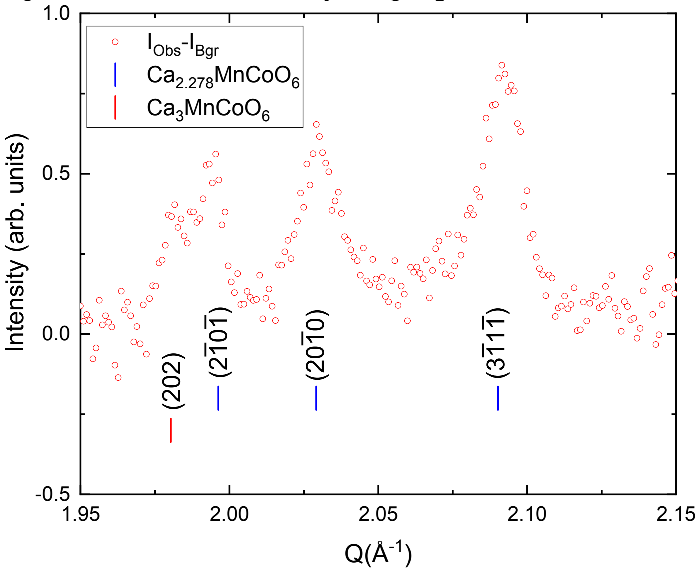

Figure S2. Region of the pattern where the most intense non-conmensurated peaks appear. The odd value of the l miller index indicates the needing of doubling c_1 in $Ca_{3-x}MnCoO_6$ with respect to $Ca_{3-x}Co_2O_6$. Pattern corresponds to the end of oxidation process.

This model (summarized in table S1) reproduces the incommensurated peaks appearing in the powder pattern, as it is illustrated in Figure S3. It is important to remark that the correct indexation of superstructure peaks renders the correct Ca content. Table S1 only reports the average atomic positions as these are the only parameters refined, when not constrained by symmetry (the fitting of modulation amplitudes would require single crystal data).

Table S1: Structure used to describe $Ca_{3-x}MnCoO_6$. The space group is $R\,3m(00\gamma)0s$ (general multiplicity 18) with a= 9.2171 Å, c_1= 4.9076 Å, and c_2=4.3387 Å ($\gamma=c_1/c_2$=1.139). Stoichiometry is then given by $(3-x)= 2\gamma$. Average atomic positions x_1, x_2 and x_3 or x_4 [$x_4= \gamma(m_3+x_3)$ within the cell placed at $m_1\boldsymbol{a}+m_2\boldsymbol{b}+m_3\boldsymbol{c}$] are given. x_{40} and Δ parameters of Crenel function indicate the center and the length (oxygen occupancy functions are centered at $x_{40}= \gamma x_3$, to warrant the synchronous change of both O1 and O2 occupancies and that Mn ions are in octahedral environment).

Atom	Site multiplicity/ Subsystem	x_1	x_2	x_3 or x_4	Occupancy or Crenel parameters
Ca	6 / 2	0.3333	0	0.25	1
Mn	3 / 1	0	0	0.018	1
Co	3 / 1	0	0	0.484	1
O1	18 / 1	0.155	0.155	0.249	x_{40}= 0.284 Δ= 0.5
O2	18 / 1	-0.155	-0.155	-0.249	x_{40}= -0.284 Δ= 0.5

Finaly, it must be noticed that the value of the propagation vector barely changes in the whole oxidation-reduction (if any) process. Incommensurated peak (against what is reported in [2]) barely move along all the process. This is illustrated in figure S3, and it indicates that the mechanism for Ca extraction in $Ca_{3-x}MnCoO_6$ is different from that in $Ca_{3-x}Co_2O_6$, being less flexible in the former. This is in accordance with the smaller Ca-diffusivity found.

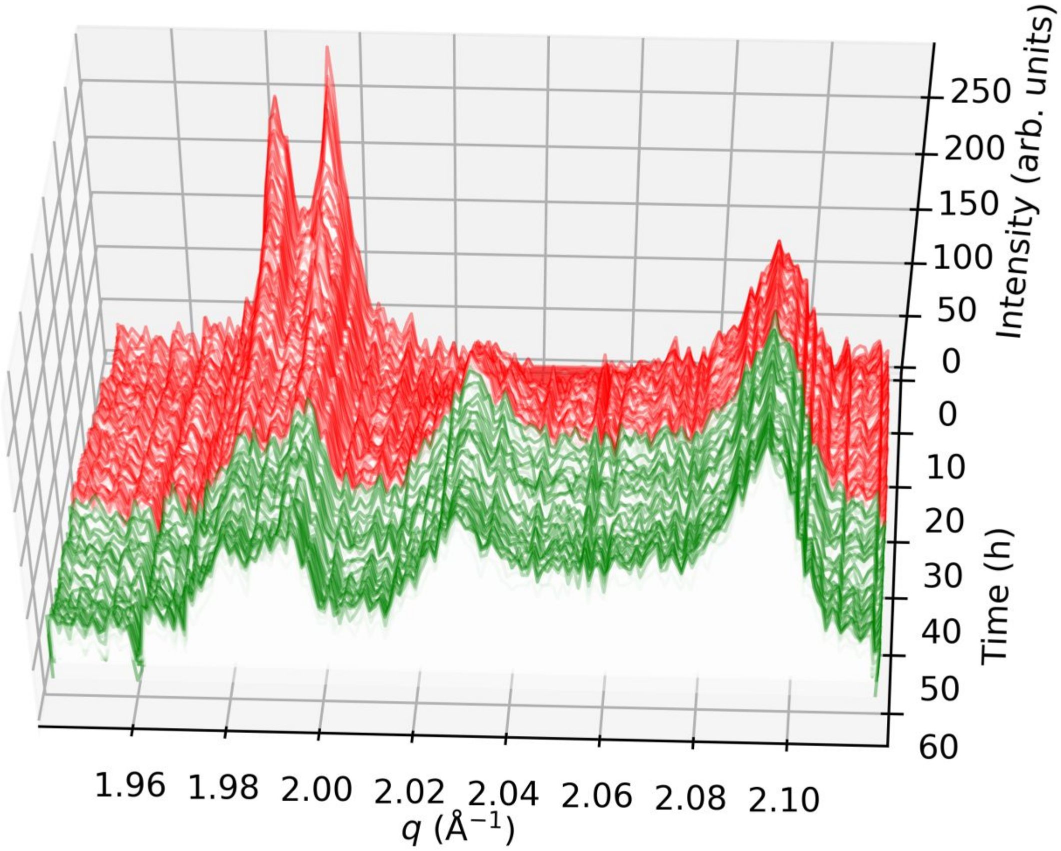

Figure S3: evolution along the oxidation-reduction cycle of the incommensurated reflections. They show that the propagation vector (and thus the Ca content of the oxidized phase) barely changes.

References:

[1] Perez-Mato, J.M., Zakhour-Nakhl, M., Weill, F. & Darriet, J. Structure of composites $A_{1+x}(A'_xB_{1-x})O_3$ related to the 2H hexagonal perovskite: relation between composition and modulation. *J. Mater. Chem.* **1999**, 9, 2795-2807.

[2] Tchitchekova, D. S.; Frontera, C.; Ponrouch, A.; Krich, C.; Barde, F.; Rosa Palacin, M. Electrochemical Calcium Extraction from 1D-$Ca_3Co_2O_6$. *Dalton Transactions* **2018**, *47*, 11298-11302.

[3] Isobe, M., Yoshida, H., Muromachi, E.T., Ohoyama, K.,Structural studies of a mixed-valence state in the incommensurate composite crystal $Sr_{1.261}CoO_3$. Sci. Technol. Adv. Mater. **2010**, *11*, 065004.

[4] Schüpp-Niewa, B., Shlyk, L., Akselrud, L., Prots, Y., Niewa, R. Crystal structure of the incommensurate modulated composite ruthenate $[Ba]_{1.30}[(Ru,Cu)O_3$
